# A Rare Case of Both Cardiac Myxoma and Left Coronary AVF, Diagnosis, and Management

**DOI:** 10.1002/ccr3.70380

**Published:** 2025-04-04

**Authors:** Isa Khaheshi, Hamed Askarpour, Seyyed Amirhossein Salehi, Atoosa Gharib

**Affiliations:** ^1^ Cardiovascular Research Center Shahid Beheshti University of Medical Sciences Tehran Iran; ^2^ Student Research Committee, School of Medicine Shahid Beheshti University of Medical Sciences Tehran Iran; ^3^ Department of Pathology, Shahid Modarres Hospital Shahid Beheshti University of Medical Sciences Tehran Iran

**Keywords:** congenital coronary arteriovenous fistula, coronary arteriovenous fistulaatrial myxoma, echocardiography

## Abstract

We present the case of a 62‐year‐old woman who was diagnosed with both cardiac myxoma and left coronary AVF, and we go on to discuss the course of her treatment and the outcome. In conclusion, this case study highlights the rare coexistence of an atrial myxoma and a congenital coronary arteriovenous fistula in a 65‐year‐old woman. A thorough cardiovascular evaluation using echocardiography and advanced imaging facilitated accurate diagnosis and informed surgical planning. The surgical approach featured the simultaneous resection of the myxoma and direct repair of the fistula, effectively minimizing surgical risks. A 65‐year‐old female presented with complaints of chest pain and shortness of breath that were exacerbated by physical activity. The results of angiography confirmed that the mass in the left atrium was indeed an atrial myxoma. Additionally, the angiography revealed a congenital arteriovenous fistula (AVF) between the left coronary arteries and the left atrium, adding another layer of complexity to the patient's condition.

1

**Summary**

- This case highlights the diagnostic and therapeutic challenges posed by the rare coexistence of atrial myxoma and congenital coronary arteriovenous fistula (AVF).
- It underscores the importance of a comprehensive diagnostic approach, utilizing advanced imaging modalities like cardiac MRI and a multidisciplinary team to optimize management.

## 2 Introduction

Primary cardiac tumors are rare, with an overall incidence rate of less than 0.1% [1]. The most common benign cardiac tumor is cardiac myxoma [2]. More than 75% of myxomas are estimated to originate in the left atrium [3]. Although non‐cancerous, cardiac myxomas can lead to significant health issues by obstructing blood flow, causing embolization, or triggering paraneoplastic syndromes [4]. Patients often present with nonspecific symptoms like dyspnea, fatigue, and syncope, complicating diagnosis [5]. Echocardiography is the primary imaging technique used to identify these tumors. Surgical excision is the definitive treatment, resulting in excellent outcomes for most patients [6].

Coronary arteriovenous fistula (AVF) is a rare vascular anomaly characterized by an abnormal connection between a coronary artery and a cardiac chamber or a receiving vein, bypassing the normal capillary network [7]. This condition can lead to significant hemodynamic alterations, including volume overload of the cardiac chambers and decreased coronary perfusion pressure [8]. While asymptomatic cases may be discovered incidentally during imaging studies, symptomatic patients can present with a range of clinical manifestations, including angina, heart failure, or arrhythmias [7]. Diagnosis often involves a combination of echocardiography, angiography, and sometimes cardiac MRI. Treatment typically requires surgical intervention to ligate the fistula and restore normal hemodynamics, reducing the risk of complications. Early detection and management are crucial in improving outcomes and enhancing the quality of life for affected individuals [9, 10].

The coexistence of atrial myxoma and congenital coronary arteriovenous fistula (AVF) is exceptionally rare, with only a few cases reported worldwide. This combination is clinically significant due to the potential for overlapping or masking symptoms, which complicate diagnosis and delays treatment. The rarity and complexity of this coexistence warrant detailed discussion to highlight the importance of a comprehensive diagnostic approach, multidisciplinary management, and the need to advance understanding of such rare cardiac conditions to improve patient outcomes. Here, we present the case of a 62‐year‐old woman who was diagnosed with both cardiac myxoma and left coronary AVF, and we go on to discuss the course of her treatment and the outcome.

## 3 Case History/Examination

A 65‐year‐old female presented with complaints of chest pain and shortness of breath that was exacerbated by physical activity. These symptoms began approximately 5–6 months before she visited the cardiologist, and they worsened in the last month.

### 3.1 Differential Diagnosis

Upon evaluation, various diagnostic tests were conducted, including an echocardiogram that revealed a large, heterogeneous, semi‐mobile mass in the left atrium, measuring 22 × 33 mm. This finding raised significant concern, prompting further investigations.

To gain a better understanding of her condition, Coronary angiography was performed (Videos 1 and 2). Coronary angiography showed tumor blush and coronary drainage (probably fistula) into the cardiac chamber (most probably left atrium).

**VIDEO 1:** Coronary angiography showed tumor blush and coronary drainage (probably fistula) into a cardiac chamber (most probably left atrium). Video content can be viewed at https://onlinelibrary.wiley.com/doi/10.1002/ccr3.70380

**VIDEO 2:** Coronary angiography showed tumor blush and coronary drainage (probably fistula) into the cardiac chamber (most probably left atrium). Video content can be viewed at https://onlinelibrary.wiley.com/doi/10.1002/ccr3.70380

The patient did not report any additional symptoms, such as fever, chills, or unintended weight loss over the past few months. There was no mention of prior medical conditions, such as hyperthyroidism, hypertension, diabetes, or other cardiovascular diseases, or specific medications that she was taking.

In her family history, the patient noted that her daughter had previously suffered from an atrial myxoma on the left side. Unfortunately, after undergoing surgical intervention for this condition, her daughter experienced severe complications resulting in brain death due to pulmonary thromboembolism.

During the physical examination, no abnormal heart sounds, murmurs, or signs of heart failure (e.g., jugular venous distension, peripheral edema) were detected, and all other examinations appeared normal. However, a notable finding was bilateral exophthalmos in her eyes. It is important to highlight that the patient did not have a documented history of hyperthyroidism, which is often associated with exophthalmos. After the diagnosis, it was decided that the best treatment for the patient was the surgical approach.

### 3.2 Operation Description

**FIGURE 1:**
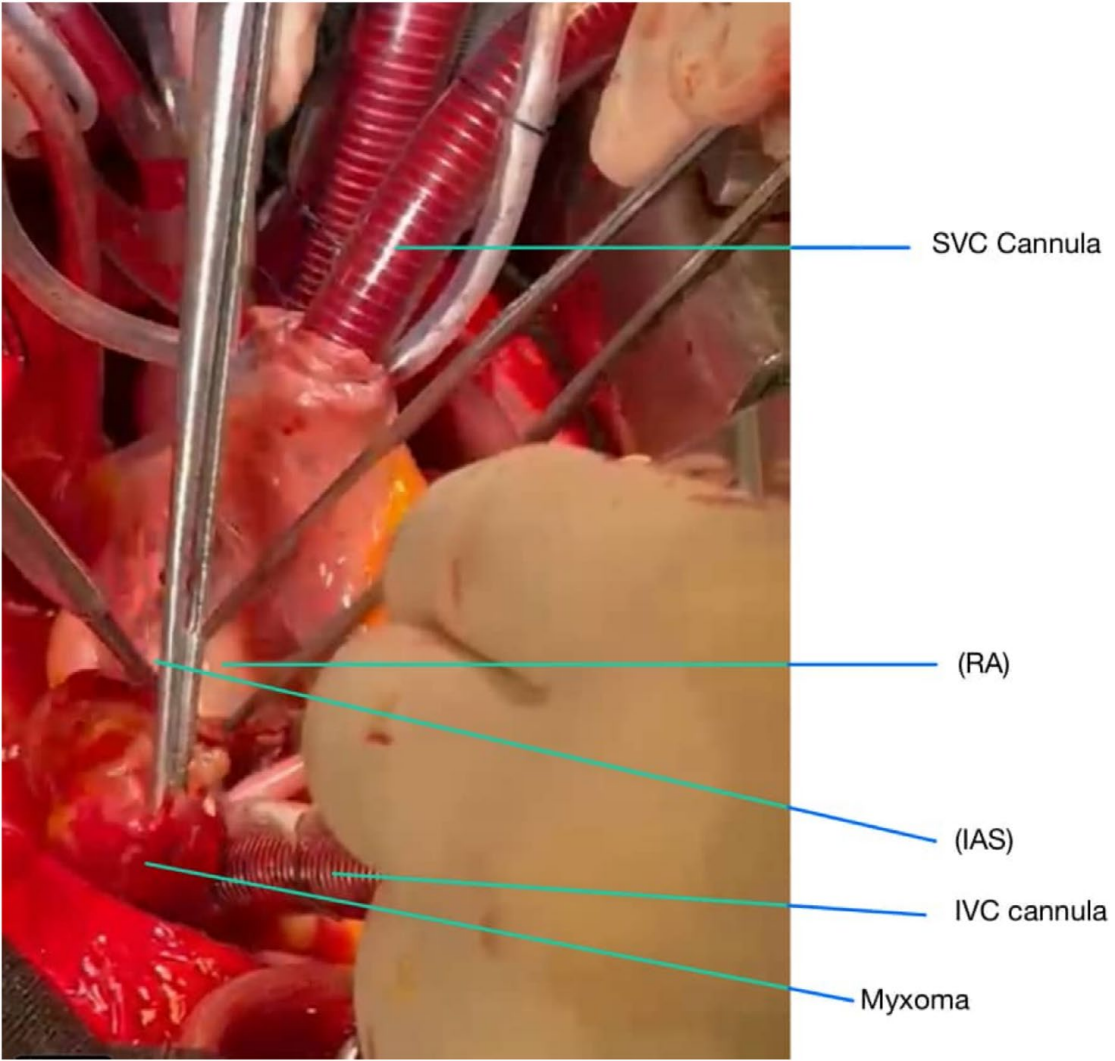
During operation the atrial myxoma in the left atrium was demonstrated.

**FIGURE 2:**
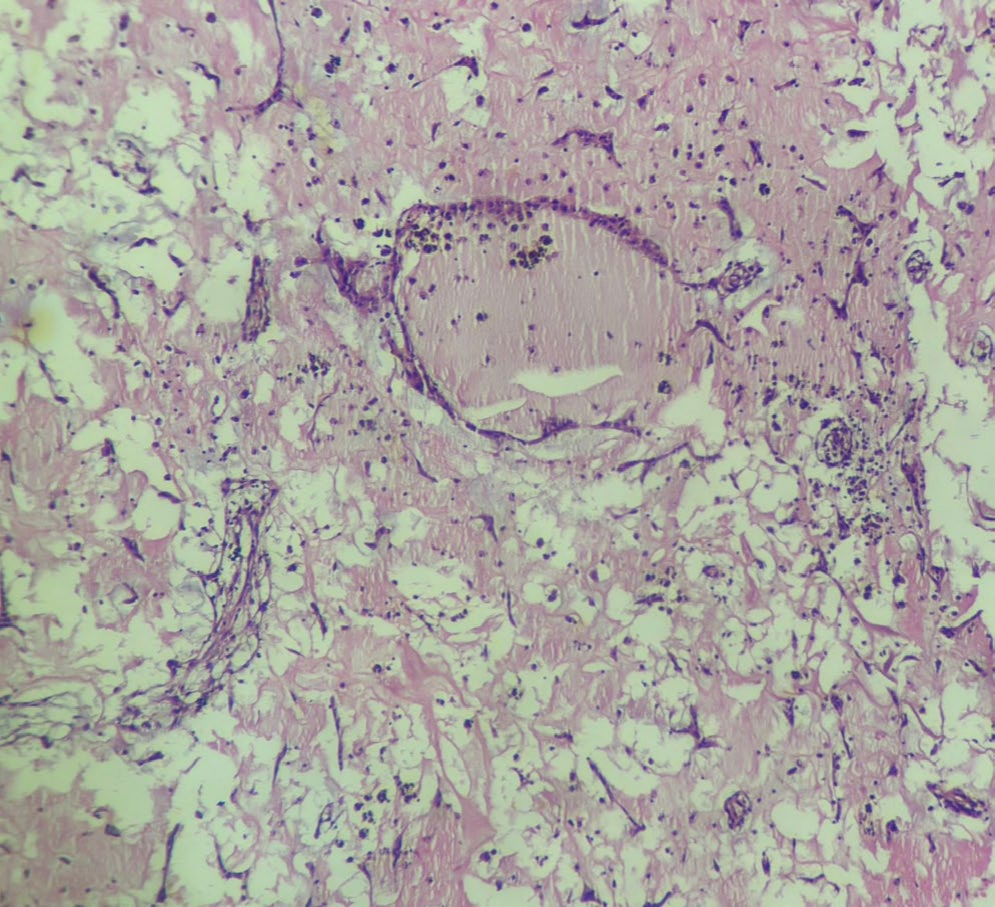
The pathology of specimen demonstrated atrial myxoma. H&E, ×40, myxoma cells around blood vessels.

**FIGURE 3:**
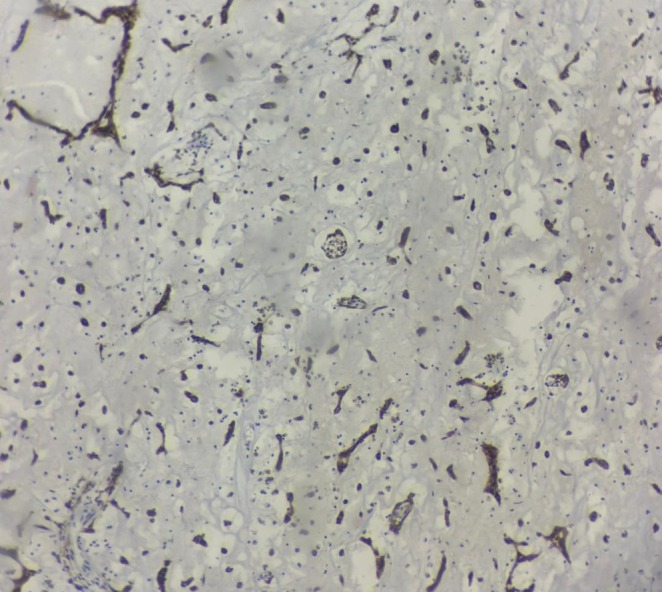
Calretinin immunostaining in myxoma cells.

**FIGURE 4:**
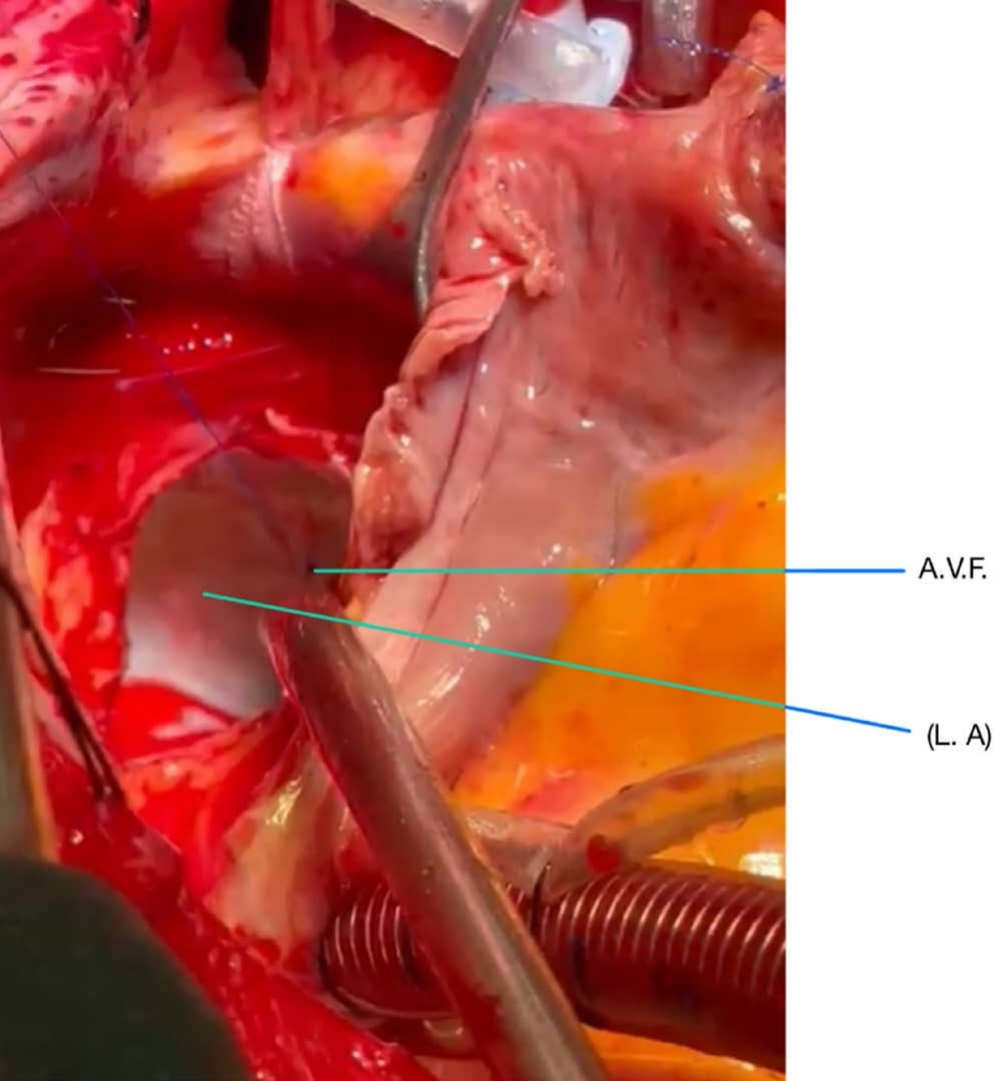
A congenital arteriovenous fistula (AVF) between the left coronary arteries and the left atrium during operation was demonstrated.

After administering general anesthesia, a median sternotomy was performed. Heparin was injected, and the pericardium was opened. Aortic and bi‐caval cannulations were performed. Cardiopulmonary bypass (CPB) was started, and the patient was cooled to 32°C. The aorta was clamped, and cardioplegic arrest was achieved. The left atrium was initially opened, revealing a substantial mass. A round mass, approximately 3 cm in diameter with a movable base, was found on the interatrial septum. After the initial examination, the inferior and superior venae cavae were clamped, and the right atrium was opened. The area of the fossa ovalis was identified, which was then incised around with a No. 11 scalpel, allowing for the complete mass removal. (Figure 1) The specimen was sent for pathology. (Figures 2 and 3) The atrial cavity was thoroughly inspected and found free of tumor particles. Subsequently, the ASD (atrial septal defect) in the fossa ovalis was repaired using a pericardial patch. Then, the AV fistula entrance into the left atrium, located posteriorly and superiorly, was over‐sewn and closed (Figure 4).

Another test with cardioplegia was performed, confirming that the situation was stable with no leaks. The patient was gradually warmed, and after closing the left atrium, the aortic clamp was released while simultaneously performing ventilation from the ascending aorta. The right atrium was closed, and the clamps around the inferior and superior venae cavae were released. Complete de‐airing was conducted, and after warming up under suitable conditions and administering a low dose of inotropes, the pump was completely turned off. The cannulas were removed. With the administration of prophylactic antibiotics and ensuring hemostasis, the sternum, fascia, and skin were closed conventionally. The patient was bandaged and transferred to the ICU with stable vital signs.

## 4 Conclusion and Results (Outcome and Follow‐Up)

The post‐op echocardiogram showed no residual mass in the left atrium, and the inter‐atrial septum was intact. The preserved systolic ejection fraction was counted at 50%–55%.

The patient was admitted to the ICU for 2 days, and then was transferred to the ward. Finally, after a total of 5‐day hospital stay, the patient was discharged with good overall general health and stable vital signs. There was a report of possible symptoms of postop complications.

Furthermore, we recommended genetic testing since there was a similar history of this condition in the patient's daughter. However, the patient refused to go forward with the test because of financial reasons.

## 5 Discussion

Cardiac myxomas are the most common primary tumors of the heart, typically arising in the left atrium [1]. These benign, gelatinous tumors can obstruct blood flow or lead to systemic embolization, with symptoms including dyspnea and palpitations. Diagnosis is usually made via echocardiography, and surgical resection is the definitive treatment [6]. Coronary arteriovenous fistulas (AVFs) are abnormal connections between coronary arteries and heart chambers or veins that can disrupt normal blood flow, potentially causing ischemia or heart failure [8]. These can be congenital or acquired and may present with symptoms like chest pain or fatigue. Diagnosis typically involves coronary angiography, and treatment often requires surgical intervention to correct the fistula [7]. Both conditions highlight the importance of thorough cardiovascular evaluation and timely management.

Here, we described the case of a 65‐year‐old woman who was referred to the cardiologist with chest pain and dyspnea on exertion starting around 6 months before she visited the doctor. After evaluations, she was diagnosed with an atrial myxoma and a congenital AVF between the left coronary arteries and the left atrium. She was then taken to surgery. The mass was removed, and the fistula was repaired. She was discharged from the hospital after complete recovery.

Several case reports have documented the occurrence of fistulas as a result of atrial myxomas. However, to date, our case stands out as the sole report of an independent incidence of an atrial myxoma coexisting with a coronary artery‐to‐atrial fistula (AVF). This unique presentation underscores the complexity and potential complications that can arise in patients with atrial myxomas.

In a recent study by Kazuhiro Suzuki et al., the authors described a case involving a left atrial mass that was reported alongside a coronary artery‐to‐left atrial fistula. In their findings, the fistula was determined to be secondary to the myxoma, with abnormal vessels extending from both the right coronary artery and left circumflex artery to the tumor situated in the left atrium. These vessels became aggregated within the tumor itself [11]. In comparison to the case by Kazuhiro Suzuki et al., where the coronary fistula was secondary to the myxoma, our case involved a congenital coronary AVF coexisting with atrial myxoma, necessitating a distinct surgical approach. Unlike their findings, our patient required simultaneous repair of the AVF and myxoma resection, highlighting the importance of tailored surgical strategies for such rare and complex conditions.

Similarly, another noteworthy report by S. Subash et al. discussed a 53‐year‐old female patient who developed an acquired coronary cameral fistula following the surgical excision of her myxoma. In this case, the authors highlighted the crucial role of transesophageal echocardiography, both during the operation and in the postoperative period, to confirm the successful closure of the fistula. Remarkably, in their patient, the fistula was repaired through the administration of protamine, a method shown in other studies to lead to the disappearance of blood flow through the fistula [12, 13]. In contrast to these reports, our case involved prior knowledge of the AVF before surgical intervention. Consequently, we opted for a proactive approach by directly over‐sewing and closing the fistula.

Although our case was successful, several points could have been approached differently to optimize care. First, comprehensive imaging with advanced modalities, such as cardiac MRI, could have provided additional insights into the anatomical relationships between the myxoma and the surrounding structures, including the AV fistula, thereby aiding in surgical planning. A multidisciplinary approach involving interventional cardiologists, cardiac surgeons, and radiologists might have optimized the management strategy for both the atrial myxoma and the congenital AV fistula. Planning a well‐defined long‐term follow‐up strategy—taking into account the patient's family history of complications following myxoma surgery—would also have been beneficial. Furthermore, recommending genetic counseling for the patient and her family may have identified hereditary syndromes that warrant surveillance for relatives, given her daughter's history of atrial myxoma.

On the other hand, this case presents several strengths that underscore its significance. The comprehensive diagnosis of both the atrial myxoma and congenitally‐associated AV fistula highlights the thoroughness of the cardiovascular evaluation, illustrating the importance of a systematic approach in patients with unusual cardiac symptoms. The surgical team's meticulous efforts in addressing both pathologies demonstrate an effective surgical strategy that reduces the patient's overall exposure to surgical risk and recovery time. The immediate pathological examination of the excised mass validates the appropriate use of surgical resources and confirms the diagnosis, reinforcing the management choices. The successful operating outcome—with no residual mass and preserved heart function—further emphasizes the effectiveness of timely surgical intervention. Lastly, this case contributes to the existing literature by documenting a rare occurrence of an atrial myxoma coexisting with a congenital AV fistula, serving as an invaluable educational resource for clinicians and raising awareness of atypical presentations associated with cardiac tumors.

Moreover, Given the patient's family history of atrial myxoma, it is strongly recommended to conduct genetic analysis or screening for hereditary syndromes, such as Carney complex or other familial cardiac tumor syndromes. Identifying potential genetic predispositions could provide valuable insights into the etiology of the condition, guide surveillance strategies for the patient and her family members, and facilitate early detection and intervention in at‐risk individuals. This proactive approach would enhance long‐term outcomes and contribute to a deeper understanding of the genetic underpinnings of such rare cardiac pathologies.

## Author Contributions

**Isa Khaheshi:** conceptualization, methodology, writing – review and editing. **Hamed Askarpour:** conceptualization, data curation, project administration, writing – original draft, writing – review and editing. **Seyyed Amirhossein Salehi:** writing – original draft, writing – review and editing. **Atoosa Gharib:** data curation.

## Ethics Statement

The authors have nothing to report.

## Consent

Written informed consent was obtained from the patient to publish this report in accordance with the journal's patient consent policy.

## Conflicts of Interest

The authors declare no conflicts of interest.

## Data Availability

“All data underlying the results are available as part of the article and no additional source data are required”.
